# Online Monitoring of Seawater Carbon Dioxide Based on an Infrared Rear Beam Splitter

**DOI:** 10.3390/s23146273

**Published:** 2023-07-10

**Authors:** Luyin Liu, Ruzhang Liu, Guochao Ma, Shanshan Feng, Yuanhui Mu, Dexi Meng, Shuying Wang, Enlin Cai

**Affiliations:** The School of Electronic Information, Qingdao University, Qingdao 266071, China; 2021023769@qdu.edu.cn (L.L.); 2021023780@qdu.edu.cn (R.L.); 2020020634@qdu.edu.cn (G.M.); fengshanshan@qdu.edu.cn (S.F.); mouyuanhui@qdu.edu.cn (Y.M.); mengdexi@qdu.edu.cn (D.M.); sy_w@qdu.edu.cn (S.W.)

**Keywords:** marine ecology, HITRAN, infrared detection, seawater carbon dioxide

## Abstract

The ocean is one of the most extensive ecosystems on Earth and can absorb large amounts of carbon dioxide. Changes in seawater carbon dioxide concentrations are one of the most important factors affecting marine ecosystems. Excess carbon dioxide can lead to ocean acidification, threatening the stability of marine ecosystems and species diversity. Dissolved carbon dioxide detection in seawater has great scientific significance. Conducting online monitoring of seawater carbon dioxide can help to understand the health status of marine ecosystems and to protect marine ecosystems. Current seawater detection equipment is large and costly. This study designed a low-cost infrared carbon dioxide detection system based on molecular theory. Using the HITRAN database, the absorption spectra and coefficients of carbon dioxide molecules under different conditions were calculated and derived, and a wavelength of 2361 cm^−1^ was selected as the measurement channel for carbon dioxide. In addition, considering the interference effect of direct light, an infrared post-splitting method was proposed to eliminate the interference of light and improve the detection accuracy of the system. The system was designed for the online monitoring of carbon dioxide in seawater, including a peristaltic pump to accelerate gas–liquid separation, an optical path structure, and carbon dioxide concentration inversion. The experimental results showed that the standard deviation of the gas test is 3.05, the standard deviation of the seawater test is 6.04, and the error range is within 20 ppm. The system can be flexibly deployed and has good stability and portability, which can meet the needs of the online monitoring of seawater carbon dioxide concentration.

## 1. Introduction

The ocean is one of the largest ecosystems on the planet and has great ecological and economic value. However, as global climate change intensifies, marine ecosystems are under increasing threat. The detection of dissolved gases in seawater plays an important role in ocean observation and exploration, which is crucial to the study of the marine environment and ecosystems. Carbon dioxide is a key factor in global warming, and seawater carbon dioxide is mainly derived from atmospheric carbon dioxide [1]. Excessive carbon dioxide entering the ocean will lead to ocean acidification, which will affect the stability and health of the marine ecosystem [2]. Therefore, it is of great scientific significance and application value to carry out online monitoring of seawater carbon dioxide.

Currently, studies on seawater carbon dioxide concentration mainly use offline sampling and analysis methods [3]. However, this method has problems such as low sampling frequency, complicated operation, and lagging results. Therefore, it is of great practical importance to carry out online monitoring of seawater carbon dioxide. Non-dispersive infrared (NDIR) gas analyzers measure the concentration of a target gas by measuring the infrared radiation absorbed by the gas. NDIR gas analyzers were invented and first used in Germany in the 1930s. Since then, thousands of infrared gas detection devices with different configurations have been produced and used in combustion, pollution, medical, automotive, chemical, refining, and other fields. In the 1960s, the US Air Force Cambridge Laboratory developed the HITRAN database and published its results to the public in 1973. The HITRAN database is one of the most comprehensive, reliable, and widely used gas molecular spectroscopy databases in the world, which is of great significance to the research of climate change, astrophysics, and other fields. Linear leaps are the main component of HITRAN, containing most of the linear transitions of molecules from the microwave to the intense ultraviolet and most of the isotopic data [4,5,6]. The most current data, published in 2020 by Gordon, Rothman, and others, include the absorption and emission properties of 51 gas molecules [7]. The HITRAN database provides a HAPI (HITRAN Application Programming Interface) for the analysis of infrared absorption characteristics of gas molecules. This interface can remotely obtain data from the database, which provides convenience for the spectral theoretical analysis of molecules. In recent years, with the development of infrared spectroscopy technology, there has been significant progress in sensors applied to the environment. Wong et al. integrated a new carbon dioxide infrared sensor into an optical fiber by using a MEMS emitter and detector [8]. The stability is improved by setting different measurement channels and reference channels, but the influence of temperature under molecular theory is ignored. Zhao et al. designed a breathing carbon dioxide sensor with a crossover cavity [9], and the influence of gas temperature on the detection effect was studied. Besson et al. reported a novel sensor based on tunable diode laser absorption spectroscopy (TDLAS) in the near-infrared region [10]. TDLAS has the advantages of [11,12] high precision and tunability. It can only be used in the near-infrared region, which is not the absorption peak area of carbon dioxide, and the laser needs to be collimated, which requires a complex hardware structure. If it is used for long-term online monitoring, both the cost and volume would be high. Vincent et al. designed a portable breath analyzer for an NDIR carbon dioxide sensor with an operating range of 0.5–4%, which greatly reduced the size of the IR carbon dioxide device [13]. Li et al. designed a seawater in situ detection device with a Raman excitation source for a polypropylene hollow fiber membrane, while the detection limit of carbon dioxide was 72.8 ppm [14]. Liu et al. developed a 4319 nm continuous-waveband inter-cascade laser (ICL) mid-infrared sensor system [15] to detect dissolved carbon dioxide in seawater, but its size and optical cost were high. Schroder et al. in 2022 proposed a new multispectral NDIR gas sensor capable of achieving a 90% decrease in signal intensity in ten seconds when the concentration was changed and set up for the direct conversion of values to ppm units [16]. In fact, most of the carbon dioxide detection is focused on air, using optical paths with multiple reflective devices [17,18,19]. It is well known that carbon dioxide molecules are polar and interact with other molecules, which affects the sensitivity and accuracy of the sensor. Tao et al. pointed out that, combined with the phase transition results in each phase and each component, more than 80% of carbon dioxide can be split in the liquid phase under controlled phase transition conditions [20], thereby achieving further carbon capture, utilization, and storage (CCUS) processes. The design of sensors needs to consider molecular theory, optical path structure, signal processing, and other aspects to ensure that the sensors can contribute to the field of environmental detection. The influence of the light interference effect and absorption coefficient is not considered, and most data fitting algorithms directly perform linear fitting on voltage signals. In the field of photoelectric sensors, Beer–Lambert’s law is an important theoretical basis for carbon dioxide detection, and few scholars have considered the impact of molecular theory. In the research of sensor systems, many systems for detecting carbon dioxide are mostly in the laboratory stage, and there are few reports on their practical applications. In view of the difficulties in the online detection of dissolved carbon dioxide in seawater, the large volume of gas detection devices, temperature drift, and other problems [14,21,22], this study proposes an online monitoring system of dissolved carbon dioxide in seawater using a single broadband light source, a thermopile detector, and coaxial membrane degassing technology, which can greatly reduce the detection cost of dissolved carbon dioxide in seawater. In addition, the system has been proven to have good stability through a comparison with NDIR commercial sensors and seawater detection. The experimental results of the entire system used for the online detection of seawater carbon dioxide show that the system can meet the needs of real-time detection and can be widely used for the online detection of dissolved carbon dioxide in liquid.

## 2. Device Principle

### 2.1. Absorption Spectrum of Carbon Dioxide Molecule

Water molecules are an important factor affecting the accuracy of infrared light detection, as in the absorption behavior of infrared light, the transitions of water molecules under infrared light can interfere with the transitions of target molecules. For the detection of dissolved carbon dioxide in seawater, the humidity of the gas in the sample will be relatively high, so it is necessary to analyze the impact of water molecules on the detection.

**Figure 1:**
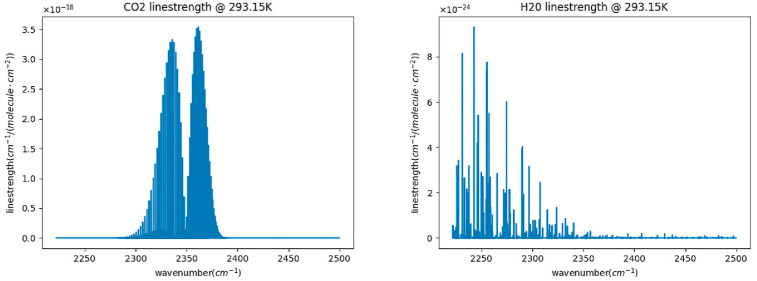
Absorption wavelength map of the HITRAN database.

**Figure 2:**
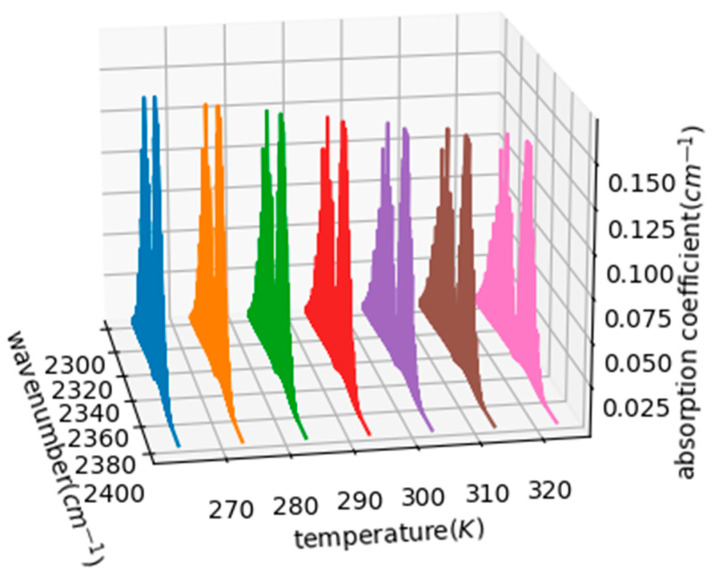
Carbon dioxide absorption coefficients at different temperatures.

We developed a calculation program for the absorption coefficient to analyze the effects of temperature, pressure, and other interfering gases on the carbon dioxide absorption coefficient. First, the program is linked to the HITRAN database, and data are obtained and stored locally according to the molecular marker, isotopic labeling, and id number in HITRAN. Next, the spectral data of water molecules and carbon dioxide molecules obtained at different wave numbers are calculated and simulated, as shown in Figure 1. It is not difficult to see from the results that in the infrared band, the absorption peak range of carbon dioxide molecules and the absorption line strength of water molecules are lower than those of carbon dioxide molecules. At 2361 cm^−1^, carbon dioxide has the highest absorption line strength of 3.542 × 10^−18^. The absorption spectrum intensity of carbon dioxide molecules is six orders of magnitude higher than that of water molecules. Therefore, designing carbon dioxide sensors with an accuracy of one part per million in seawater environments can have good resolution. Then, the carbon dioxide molecular absorption coefficient based on the Voigt spectral line profile is calculated.

(1) $$f_{v}=\frac{\gamma \left(p,T\right)\ln2}{\pi \alpha_{D}^{2}}\int_{-\infty }^{+\infty }\frac{e^{-t^{2}}}{{\left(\frac{\gamma \left(p,T\right)}{\alpha_{D}}\right)}^{2}\cdot \ln2+{\left(\frac{\nu -\nu_{0}}{\alpha_{D}}\sqrt{\ln2}-t\right)}^{2}}dt,$$

where *γ* is the Lorentzian half-height width; $\alpha_{D}$ is the Doppler half-height width; and *T* and p are temperature and air pressure, respectively. It is known from Lambert’s law that changes in the absorption coefficients of carbon dioxide affect the accuracy of the concentration measurements. The Voigt line pattern can provide important spectral information, such as line width, offset, and intensity. The actual spectral line pattern is closest to the Voigt line pattern, which is the convolution of the Doppler and Lorentz line pattern. Usually, the environment temperature of seawater varies from 263.15 K to 323.15 K (−10 °C~50 °C), and the atmospheric pressure in shallow seawater is generally 1 atm. The program analysis wavenumber is set in the waveband of two absorption peaks of carbon dioxide molecules, between 2300 cm^−1^ and 2400 cm^−1^. Then, the temperature of the program is set to 263.15 K, 273.15 K, 283.15 K, 293.15 K, 303.15 K, 313.15 K, and 323.15 K, and standard atmospheric pressure is used to obtain the carbon dioxide absorption coefficient between 2300 cm^−1^ and 2400 cm^−1^ at different temperatures, as shown in Figure 2.

According to the data analysis results, the absorption coefficient of carbon dioxide molecules decreases with increasing temperature. On the one hand, as the temperature increases, the thermal energy of molecular vibration increases and the absorption peaks of most bands will redshift—that is, moving to the long wave direction—and new spectral lines will also appear, which will cause the absorption coefficient to decrease. On the other hand, as the temperature increases, the distance between the molecules increases, the interaction between the molecules decreases, and the width of the absorption line increases, so the absorption coefficient decreases. In practical applications, it is necessary to control the temperature of the system. It is also necessary to consider setting the reference channel and the measurement channel for differential detection, and perform differential fitting of the two pairs of voltage signals, to perform system-specific correction of Beer’s law [23].

(2) $$C=\frac{1}{kL}\ln\left[\sum_{i=0}^{N}a_{i}{\left(\frac{U_{0}}{U}\right)}^{i}\right]$$

where U denotes the outgoing light intensity, $U_{0}$ denotes the incident light intensity, k denotes the absorption coefficient for a particular combination of gas and filter, L denotes the optical path length, and the C denotes the concentration of the target gas. i is the optimal solution to the polynomial fit under the least squares criterion, determined from the calibrated measurements, and $a_{i}$ are the coefficients of the individual polynomial fit.

### 2.2. Optical Path Design and Simulation

The optical path is an important component of photoelectric sensors. A post-spectroscopic structure was considered in order to avoid the interference effects of infrared detectors. The method was realized by a beam splitter, which has high reliability and can ensure the accuracy and reliability of long-term use.

In this study, TracePro was used for the optical simulation of the design of the rear beam splitter structure. Firstly, the beam splitting structure was modeled by placing the IR beam splitter at forty-five degrees and setting the material to be a single crystal of calcium fluoride, with one surface coated with a beam-splitting film and a forty-five-degree incident beam splitting ratio (R/T) of 1 and another surface coated with a transmission-enhancing film. Then, two detector panels were deployed with specific wavelengths to simulate the effect of filters, one at 4.23 µm and the other at 3.95 µm. A 30-ring light source with an outer radius of 15 mm and an inner radius of 0 was set up for the grid point source. The two detectors were adjusted several times so that they were at the same distance from the beam splitter and simulated.

**Figure 3:**
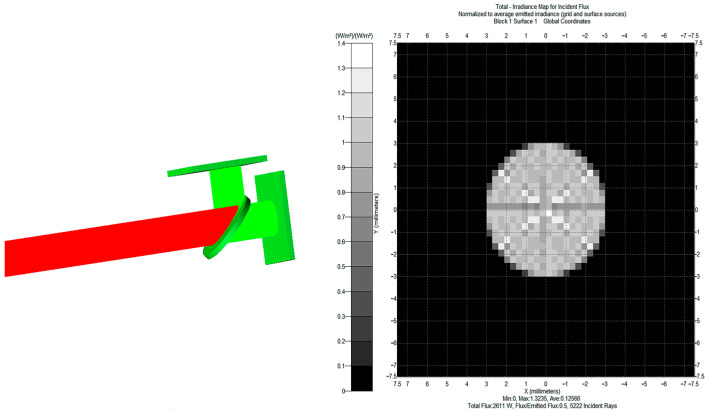
Post-split optical path diagram.

Based on the above setup information, the infrared light ray tracing simulation was performed. The optical path is shown in the diagram. Both paths were detected at equal distances from the center of the beam splitter and on the same centerline. The irradiance diagram is shown in Figure 3 with a minimum value of 0, a maximum value of 1.3235, an average value of 0.12566, a total flux of 2611 W, a flux/emission flux of 0.5, and 5222 incident rays. The infrared light is divided into two channels by the beam splitter and irradiated on two detection panels. As shown in Figure 3, the irradiation images of the two detection plates are consistent through multiple experimental adjustments. The illumination map of light is relatively uniform, which can well meet the working conditions of the thermopile detector to obtain more stable detection signals.

## 3. System Structure and Methods

In the previous section of the molecular spectrum, the result showed that the absorption peak of carbon dioxide molecules at 2361 cm^−1^ was 3.542 × 10^−18^ molecules/cm^2^. This was the basis for our selection of the measurement point and reference point. Therefore, a narrow-band filter with a center wave number of 2361 cm^−1^ was selected at the measurement end, and a filter with a center wave number of 2531 cm^−1^ was selected at the reference end. The filters were placed in front of the thermopile detector, respectively, to prevent the interference of light in other frequency bands. In practical applications, it is necessary to consider the gas–liquid separation structure and signal conditioning method, which will affect the cost and detection performance of the device.

### 3.1. Device Structure

**Figure 4:**
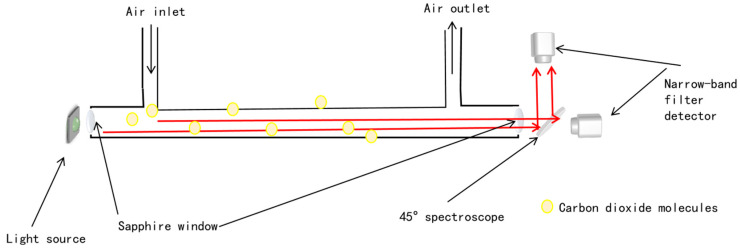
Schematic diagram of post-infrared spectroscopy.

**Figure 5:**
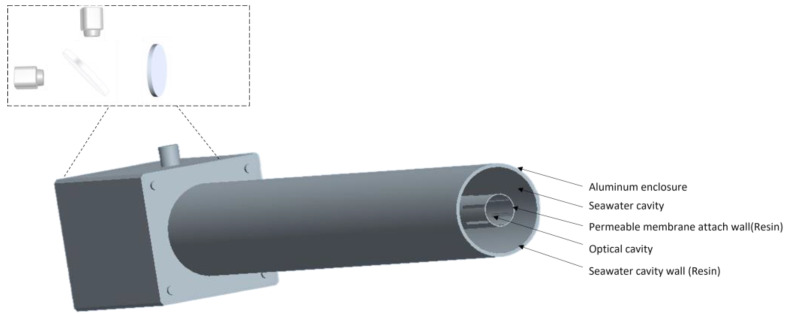
Schematic diagram of the post-infrared spectroscopic structure. The seawater and gas chambers are separated by a degassing film attachment wall, in front of the detector, a forty-five-degree fixed IR spectroscope, and a protective CaF_2_ window.

**Figure 6:**
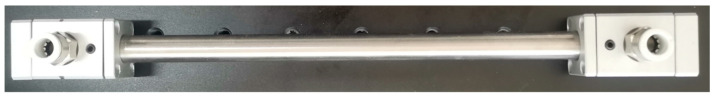
Structure of the infrared detection chamber.

Figure 4 depicts the schematic diagram of the post-infrared spectroscopic structure. As shown in Figure 5, when seawater enters the seawater cavity, it contacts with the permeable membrane, and the molecular diffusion of carbon dioxide diffuses into the optical cavity. Figure 6 shows the actual structure. Due to the influence of temperature on the absorption coefficient of carbon dioxide molecules, it is necessary to control the system at a constant temperature. The temperature control adopts a PTC constant-temperature electrothermal film wrapped on the external pipe wall. This material has self-limiting current characteristics, that is, under a certain voltage, the current size will be automatically adjusted to ensure the stability of the surface temperature of the membrane material.

When carbon dioxide gas held in seawater passes through the infrared detection chamber, the carbon dioxide gas molecules absorb infrared light at specific wavelengths. If two filters are placed on the same optical path, light with different wavenumbers will interfere, resulting in an increased detection error. Therefore, we carried out light separation processing. An infrared spectroscope is an optical device commonly used to separate radiation of different wavelengths within the infrared spectral range and focus them on the detector. It has high transmittance, which can reduce reflection and scattering when light passes through and improve the efficiency of light transmission. The beam splitter is used at an angle of forty-five degrees. One side is coated with a beam-splitter film and the other side is coated with a transmission enhancement film so that it can divide the incident light into two parts: reflected light and transmitted light. The beam splitter is light in weight, reasonable in price, and relatively easy to produce. According to the wavelength and direction of polarization, they can be used in optical instruments, electrochemical and medical instruments, and other photoelectric sensing devices, in which light can be divided according to different light intensity ratios. Here, the infrared spectrum is refracted at an angle of forty-five degrees so that the transmitted light and the reflected light (S: 50%, P: 50%) are the same, and the position of the thermopile detector is calculated to ensure the two optical paths are the same. One reaches the detector through the 4.23 µm filter and the other reaches the other detector through the 3.95 µm filter. The two-channel differential detection has a good effect on reducing environmental interference and eliminating noise. The infrared light emitted by the light source propagates in all directions, and its intensity decays after passing through the reflecting surface and gas absorption. Part of the light is reflected too many times, and the time it takes to reach the sensitive surface of the probe results in negligible signal strength. The light that has been reflected multiple times is not an effective incident light, so this system design adopts a direct beam optical path structure, which can reduce the cost of system design and also reduce optical noise. According to Jones [24], there are three different types of noise in a thermal detector: temperature noise, thermal noise, and signal noise. The first two types are the main noise in thermopile detectors [25]. MEMS thermopiles are very sensitive to temperature variations. For the selection of a thermoelectric detector, the dipole moment per unit volume of spontaneous polarization in a unit volume of material is expressed as the amount of charge per unit area perpendicular to the direction of spontaneous polarization. On the surface insulating layer of a thermoelectric material, the thermoelectric material is absorbed by the surface charge through spontaneous polarization and neutralization. The total amount of spontaneous polarization is influenced by changes in temperature due to the irradiation of infrared light on the surface of the material. When the neutralization state between the surface charge and the adsorbed ionic charge is broken, the relaxation time of the surface charge of the sensing element changes, breaking the electrical equilibrium and generating an additional charge. The spontaneous polarization of the surface charge therefore varies with temperature, a phenomenon often referred to as the pyroelectric effect.

**Figure 7:**
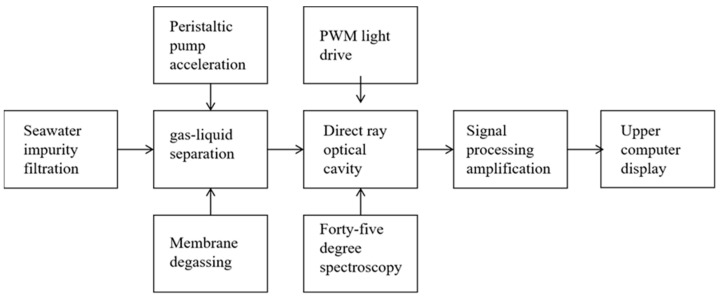
Block diagram of the seawater CO_2_ online monitoring system.

The system configuration used for seawater-dissolved carbon dioxide: Based on the infrared post-spectroscopic technology, a seawater carbon dioxide online monitoring system was designed and its system block diagram is shown in Figure 7.

The system consists of a gas–liquid separation module, an infrared spectral analysis module, a computer control module, and a data processing module. As seawater contains plankton and a large number of solid impurities, it is necessary to carry out a series of treatments on seawater. First, porous filter stones are placed at the water inlet to isolate solid impurities and prevent them from interfering with the seawater sample. In the gas–liquid separation module, we use a peristaltic pump to accelerate the flow of seawater sample in the pipeline. On the one hand, this can accelerate the gas–liquid separation efficiency; on the other hand, it can slow down corrosion from the long-term contact of seawater with the pipeline. Polytetrafluoroethylene degassing film is an inert material with good acid resistance, alkali resistance, and corrosion resistance. It can be used as a degassing film for gas–liquid separation of seawater. Next, carbon dioxide enters the optical detection cavity through the degassing membrane. When infrared light passes through carbon dioxide molecules, the specific frequency of infrared light is absorbed by them. The detector receives the infrared light absorbed by the gas and converts it into electrical signals. Finally, the detected electrical signals are collected by the AD chip, filtered, analyzed, calculated, and then sent to the host computer for display.

### 3.2. Experimental System

**Figure 8:**
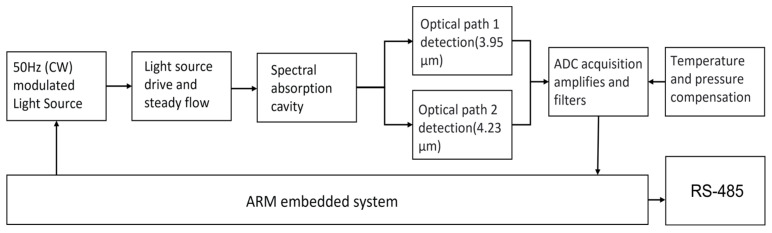
Schematic diagram of detection.

**Figure 9:**
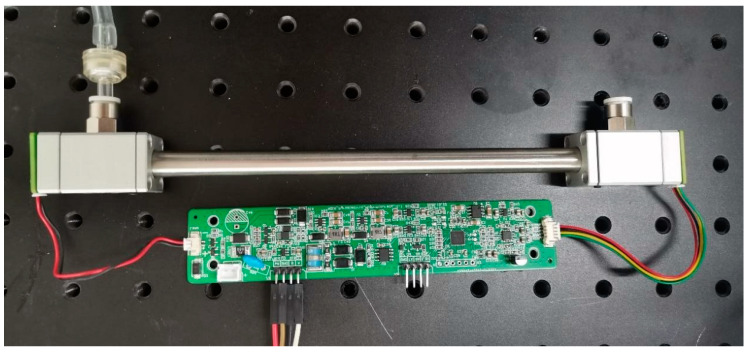
Optical equipment for experiments.

As the core part of the system, the circuit provides a guarantee for the light source module, detector module, and signal processing transmission. In the design, the constant output of light source intensity requires a stable input voltage and current. Figure 8 is the flowchart of the circuit system. The system uses PWM (pulse width modulation) technology and connects the triode and diode to adjust the light source stably. In order to achieve a faster response, an independent high-speed A/D conversion chip is selected. It has a high-precision 24-bit analog-to-digital converter that can achieve weak signal acquisition, perfectly combining DC accuracy and AC performance. Considering the accuracy and stability of data processing, high-precision electronic components are selected in the circuit board, and feedback circuits are used to amplify and stabilize the sampled signals. After filtering and amplifying the sampled signals of the two channels, they are sent to the MCU for inversion, and the concentration value of carbon dioxide detection is obtained. In addition, an RS-485 communication mode is set up to communicate with the host computer. This mode adopts differential level signal transmission, which has a strong anti-interference ability and long transmission distance. The hardware cost of the 485 communication protocol is relatively low, which is suitable for large-scale applications and low-cost application scenarios. The MCU controls the operation of the whole system. In order to reduce the area of the circuit board, we adopt the method of multi-layer wiring to arrange the board. The actual overall structure is shown in Figure 9.

The system is designed to be included in mobile devices, with size and cost being key factors. In the coaxial detection cavity, the infrared detection module includes a brand infrared light source, which is a micromechanical thermal infrared transmitter with high emissivity and long life. It provides true blackbody radiation characteristics of 2 to 14 microns and has a high radiant flux near 4 microns, which can be well applied to the detection of infrared absorption materials near this wavelength. The EMIRS200 is packaged in TO-39. It is a three-pin light source with a CaF_2_ window. It has high transmittance in the infrared band, which can reduce the scattering in the optical system and improve the system performance. At the same time, the CaF_2_ window has good corrosion resistance, and can still maintain good performance in harsh environments such as strong acid and strong alkali. In order to accelerate the flow of gas and respond more quickly to changes in concentration, the gas is then purged at the gas inlet, thus accelerating the flow of gas and increasing the sensitivity of the system. The forty-five-degree beam splitter is placed at the end of the gas chamber to separate the optical path, and two detectors are selected and placed in two positions. The broad-spectrum infrared light generated by the wavelength scan is absorbed by the gas in a specifically shaped spectral line pattern, and the electrical signal converted by the infrared detector produces a corresponding shaped depression called the absorption signals.

After determining the direct light distance of 24 cm, according to the absorption coefficient calculated in the previous absorption spectrum of carbon dioxide molecules, the absorption coefficient is calculated to be 0.1494 cm^−1^ at 20 °C by the HITRAN database. Then, the system is calibrated according to the actual measured voltage value. During the calibration, the equipment is powered on, the electric heating sheet is turned on for constant-temperature control and peristaltic pump acceleration, and a series of experiments are carried out at an ambient temperature of 20 °C and standard atmospheric pressure, that is, a series of liquid samples with different concentrations are prepared, with an interval of 100 ppm. The standard liquid is pumped into the experimental equipment. After waiting for the signal from the reference circuit ($U_{0}$) and the signal from the measurement circuit (U) to stabilize, the concentration versus ratio obtained at different concentration points ($\frac{U_{0}}{U}$) is recorded, based on the logarithmic ratio of the actual carbon dioxide concentration to the voltage of the reference and measurement channels $\ln\left(\frac{U_{0}}{U}\right)$. Multi-point recording and optimal polynomial coefficient fitting are performed to correct for Beer’s law and to convert the units of concentration directly to parts per million (ppm) with a resolution of parts per million.

## 4. Results Analysis and Discussion

At present, most of the carbon dioxide detection systems are used to measure the gas in the atmosphere, but there is a lack of detection of carbon dioxide in seawater. The system is compared with the current commercial NDIR gas sensor. As commercial sensors have been integrated, numerical reading is relatively slow and lacks some flexibility. The hardware in this study is independently developed. The data conversion efficiency and numerical reading in the system can be flexibly set by the MCU in the lower computer, which provides solution support for online detection technology.

**Figure 10:**
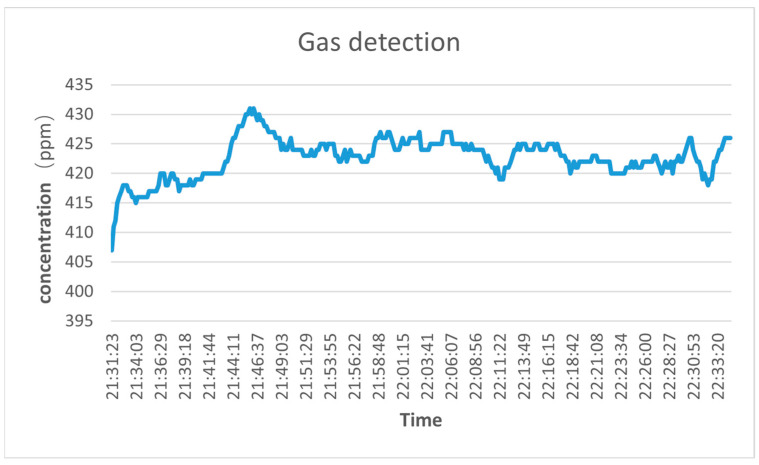
Gas environment detection test.

In order to evaluate the performance of the seawater-dissolved carbon dioxide detection system designed in this paper, the system is tested in the atmosphere and shallow seawater. Firstly, the stability of the sensor system is tested to verify that it can meet the needs of online detection. The equipment is sealed in a closed container, and the power line and signal line are extracted through the flange port. The gas detection temperature is 293.15 K and the pressure is 1 atm. Through multiple measurements using NDIR gas sensors, the concentration of carbon dioxide in the atmosphere is 420 ppm, the sampling time is ten seconds, and the continuous online monitoring time is one hour. The gas test environment is selected in an open outdoor environment to avoid the impact of breathing and industrial activities on the test. The test results are shown in Figure 10.

**Figure 11:**
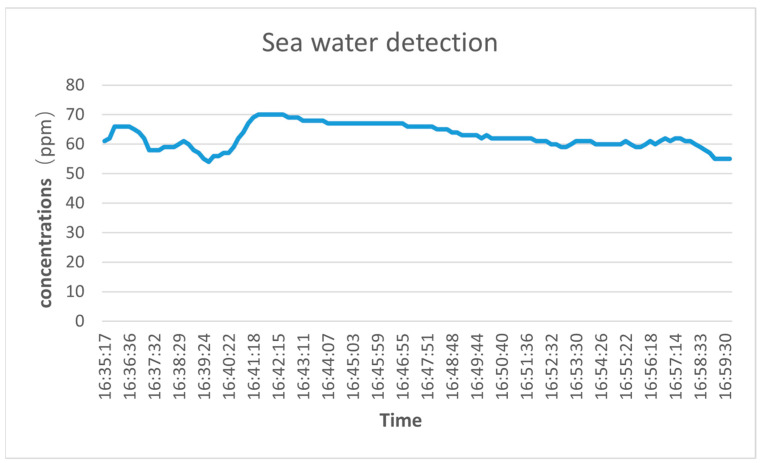
Seawater carbon dioxide detection test.

The experimental results show that the mean value of the system in the air is 422.8239 ppm, and the standard deviation is 3.05, which has a good test effect. The accuracy of this measurement is about 5% and the relative error with the true value is 0.69%. It has good accuracy and stability, and the stability error range is within 20 ppm, which can meet the needs of online monitoring. Compared to the general accuracy of commercial gas sensors ranging from 2% to 10%, the accuracy of this system is within this range. Therefore, the system can meet the needs of real-time measurement in the gas environment. Concentration conversion mostly uses polynomial fitting based on molecular theory, which has better robustness and stability than the linear fitting method. Generally speaking, the structure constructed by this system can also be suitable for the detection of other molecules with infrared absorption characteristics. MCU sampling is usually completed in an instant, allowing for more flexible changes in the system’s response rate. In addition, the results show that the concentration of carbon dioxide in the atmosphere has exceeded the historical average and continues to increase. This situation may lead to global warming, sea level rise, climate change, and other issues. Therefore, we should take actions to reduce carbon dioxide emissions, such as improving energy efficiency, using renewable energy, and promoting low-carbon lifestyles. In addition, stability tests were performed in seawater, as illustrated in Figure 11.

In general, the content of carbon dioxide in seawater is 2.2 mmol/kg. Based on this value, we convert its content to approximately 96.8 ppm and evaluate the performance of the system.

Due to the difference between the seawater and atmospheric environments, there are large fluctuations in the initial placement of the device in seawater, including but not limited to the effects of circuit warm-up and seawater flow on the fluctuations of the device. The data show that the in situ detection of seawater fluctuates within 20 ppm, with a standard deviation of 6.04, indicating good stability. Although the concentration measured by the system is slightly lower than the dissolved carbon dioxide content in the ocean, it exhibits good stability. The lower value of carbon dioxide in shallow seawater than in the atmosphere is due to the presence of a large number of microorganisms in the seawater that undergo chemical reactions. Organisms in the ocean absorb carbon dioxide through photosynthesis, convert it into organic matter, and release oxygen. In addition, there are other chemical processes in seawater, such as the dissolution and deposition of carbonate rocks, which can also affect the concentration of carbon dioxide in seawater. Therefore, the concentration of carbon dioxide in shallow seawater is relatively low. However, due to the limitations of the Teflon waterproof and breathable membrane, the system still needs further optimization and improvement for the high-pressure environment of the deep ocean to adapt to the more complex and variable marine environment. In addition, the experimental device can detect carbon dioxide content in rivers, lakes, oceans, and other liquids, and can be extended to add pH, salinity, dissolved oxygen, methane, and other indicators on the basis of carbon dioxide detection to meet different detection target requirements. Due to the low cost and portability of the device, array detector units can be deployed in the future for multi-component dissolved gas detection.

## 5. Conclusions

This study proposes a new type of system for the real-time monitoring of seawater carbon dioxide, which has flexibility, portability, high stability, and good accuracy, and can be used for the online monitoring of seawater. The system of seawater-dissolved gas detection starts from molecular theory and, based on the simulation results of the carbon dioxide infrared absorption spectrum, the absorption line strength of carbon dioxide molecules in the mid-infrared band is six orders of magnitude higher than that of water molecules. Therefore, detection at parts per million can be achieved in this band. At the same time, considering the interference of light, the infrared rear beam-splitter method is used to design the optical path structure. This system includes a coaxial seawater gas–liquid separation structure, peristaltic pump to accelerate the water flow, a direct infrared detection structure, polynomial ratio voltage fitting to modify Beer’s law, and the hardware design of a low-cost, high-precision, and stable acquisition system. The system has important significance and application value and can provide important technical support for marine ecological protection and management. Compared with traditional chemical analysis methods, this system has the advantages of fast detection speed, simple operation, low cost, and high real-time performance, and it is more portable and flexible because it does not require additional consumables and only requires power supply. It can be widely used in marine environmental monitoring and marine ecological research. Through the analysis of experimental data, this paper also explores the law of pressure, temperature, and other changes on carbon dioxide, which provides a reference for further research on the operation mechanism of the marine ecosystem. The experimental results show that the system has the advantages of high sensitivity, high resolution, and high stability, and can meet the needs of online monitoring of seawater carbon dioxide concentration.

In summary, the system of seawater-dissolved gas detection designed in this paper is based on molecular theory for optimal polynomial fitting to modify Beer’s law and is tested in seawater, with high detection accuracy and stability, which can meet the needs of marine environmental monitoring and scientific research.

## Data Availability

Not applicable.
